# Effective continuing professional development for translating shared decision making in primary care: A study protocol

**DOI:** 10.1186/1748-5908-5-83

**Published:** 2010-10-27

**Authors:** France Légaré, Hilary Bekker, Sophie Desroches, Mary Politi, Dawn Stacey, Francine Borduas, Francine M Cheater, Jacques Cornuz, Marie-France Coutu, Norbert Donner-Banzhoff, Nora Ferdjaoui-Moumjid, Frances Griffiths, Martin Härter, Cath Jackson, André Jacques, Tanja Krones, Michel Labrecque, Rosario Rodriguez, Michel Rousseau, Mark Sullivan

**Affiliations:** 1 Research Center of Centre Hospitalier Universitaire de Québec, Hospital St-François D'Assise, Knowledge Transfer an Health Technology Assessment Research Group, 10 Espinay, Québec, QC, G1L 3L5, Canada; 2 Leeds Institute of Health Sciences, School of Medicine, Charles Thackrah Building, University of Leeds, 101 Clarendon Road, Leeds, LS2 9LJ, UK; 3 Health Communication Research Laboratory, George Warren Brown School of Social Work, Washington University in St. Louis, Campus Box 1009, 700 Rosedale Ave St. Louis, MO 63112-1408, USA; 4 School of Nursing, Faculty of Health Sciences, University of Ottawa, Guindon Hall, 451 Smyth Road, Ottawa, ON, K1H 8M5, Canada; 5 Continuing Professional Development Office, Faculty of Medicine, Université Laval, Pavillon Vandry, Cité Universitaire, Québec, QC, G1K 7P4, Canada; 6 Institute for Applied Health Research, Glasgow Caledonian University, Cowcaddens Road, Glasgow, G4 0BA, UK; 7 Department of Ambulatory Care and Community Medicine & Clinical Epidemiology Centre, University of Lausanne, Bugnon 44, Lausanne, CH-1011, Switzerland; 8 Centre for Action in Work Disability Prevention and Rehabilitation, Rehabilitation Department, Université de Sherbrooke, Longueuil, 1111, St-Charles West, room 101 Longueuil, QC, J4K 5G4, Canada; 9 Department of General Practice and Family Medicine, Philipps-Universität Marburg, Allgemeinmedizin, Präventive und Rehabilitative Medizin, Karl-von-Frisch-Straße 4, D-35043 Marburg, Germany; 10 Centre Léon Bérard, Université de Lyon, 28 Rue Laennec, 69008 Lyon, France; 11 Health Sciences Research Institute, Warwick Medical School, University of Warwick, Coventry, CV4 7AL, UK; 12 Institut und Poliklinik für Medizinische Psychologie, Zentrum für Psychosoziale Medizin, Universitätsklinikum Hamburg-Eppendorf, Martinistrasse 52 (Gebäude W 26) D-20246 Hamburg, Germany; 13 School of Healthcare, University of Leeds, Baines Wing Leeds, LS2 9UT, UK; 14 Practice Enhancement Division, Collège des médecins du Québec, 2170, boulevard René-Lévesque West, Montreal, QC, H3H 2T8, Canada; 15 Institute of Biomedical Ethics, Centre for Ethics of the University of Zurich, Pestalozzistrasse 24 CH-8032, Zurich, Switzerland; 16 Department of Family Medicine, Faculty of Medicine, McGill University, Pine 517 Montreal, QC, H2W 1S4, Canada; 17 Departement of Family Medicine and Emergency Medicine, Université Laval, Pavillon Vandry, Cité Universitaire, Québec, QC, G1K 7P4, Canada; 18 Department of Psychiatry and Behavioral Sciences, University of Washington, Box 356560, Seattle, WA 98195, USA

## Abstract

**Background:**

Shared decision making (SDM) is a process by which a healthcare choice is made jointly by the healthcare professional and the patient. SDM is the essential element of patient-centered care, a core concept of primary care. However, SDM is seldom translated into primary practice. Continuing professional development (CPD) is the principal means by which healthcare professionals continue to gain, improve, and broaden the knowledge and skills required for patient-centered care. Our international collaboration seeks to improve the knowledge base of CPD that targets translating SDM into the clinical practice of primary care in diverse healthcare systems.

**Methods:**

Funded by the Canadian Institutes of Health Research (CIHR), our project is to form an international, interdisciplinary research team composed of health services researchers, physicians, nurses, psychologists, dietitians, CPD decision makers and others who will study how CPD causes SDM to be practiced in primary care. We will perform an environmental scan to create an inventory of CPD programs and related activities for translating SDM into clinical practice. These programs will be critically assessed and compared according to their strengths and limitations. We will use the empirical data that results from the environmental scan and the critical appraisal to identify knowledge gaps and generate a research agenda during a two-day workshop to be held in Quebec City. We will ask CPD stakeholders to validate these knowledge gaps and the research agenda.

**Discussion:**

This project will analyse existing CPD programs and related activities for translating SDM into the practice of primary care. Because this international collaboration will develop and identify various factors influencing SDM, the project could shed new light on how SDM is implemented in primary care.

## Background

### The importance of addressing decision making in primary care

Primary health care can be defined as the 'level of a health service system that provides entry into the system for all new needs and problems, provides person-focused (not disease-oriented) care over time, provides care for all but very uncommon or unusual conditions, and coordinates or integrates care provided elsewhere or by others' [1]. Countries with a strong primary healthcare system can improve their populations' health outcomes and are better able to avoid excessive health services costs [2,3].

Two studies from the United States have shown the increased importance of primary health care. One study found that on average, 800 out of 1,000 individuals experience medical symptoms every month. Of those 800, 327 consider seeking medical care and most visit a primary care physician [4]. Additionally, the American Medical Association Physician Socioeconomic Statistics (2003) showed that most medical consultations are performed by primary care physicians [5]. Together, these data emphasize the importance of addressing decision making in primary care, the sector in which most individuals seek health-related advice [4].

### The importance of translating shared decision making into primary care

Growing numbers of stakeholders agree that financial, organisational, and quality-related problems that menace healthcare systems around the world require change in the way that patients are engaged as partners in their health care [6]. Shared decision making (SDM) is an interactive process by which patients and practitioners collaborate in choosing health care. A systematic review identified 31 distinct SDM components and summarized key elements in an integrative model [7,8]. In this model, SDM is achieved by knowing and understanding the best available evidence on the risks and the benefits of every available option, while considering patients' values and preferences [9-11]. More specifically, based on the integrated model proposed by Makoul and Clayman (2006), SDM comprises the following essential elements: defining/explaining the problem, presenting the options, discussing the pros/cons (benefits/risks/costs), exploring the patient's values/preferences, discussing the patient's ability/self-efficacy, presenting the doctor's knowledge/recommendations, checking/clarifying the patient's understanding of the issue, making or explicitly deferring a decision, and arranging follow-up [7]. Policy makers see SDM as desirable because of its potential to reduce the overuse of options unclearly associated with benefits (*e.g*., prostate cancer screening) [12]; enhance the use of options clearly associated with benefits (*e.g*., cardiovascular risk factor management) [13]; reduce unwarranted healthcare practice variations [14]; and foster the sustainability of the healthcare system from a health policy maker's perspective [15].

A significant proportion of patients prefer to take active role in making decisions concerning their health, especially once they understand the implications of doing so [16]. Notably, patients' active participation in decision making is associated with favourable health outcomes [17,18]. Modifying barriers that patients perceive as impeding them from sharing decisions with their healthcare professional makes it more likely that patients will embrace a more active role. By extension, enabling health professionals to explicitly translate SDM into clinical practice may benefit patients' healthcare experience and treatment. Nonetheless, primary care practitioners have not yet been widely adopting SDM [19,20].

### The importance of continuing professional development to knowledge translation

Knowledge translation (KT) is defined as 'a dynamic and interactive process that includes synthesis, dissemination, exchange, and ethically sound application of knowledge to improve the health of individuals, provide effective health services and products, and strengthen the healthcare system' [21]. The knowledge-to-action process conceptualizes the relationship between knowledge creation and action, with each concept comprising ideal phases or categories [22]. The knowledge creation 'funnel' conveys the idea that knowledge needs to be adapted before it can be applied in clinical contexts. The action part of the process can be thought of as a cycle leading to the implementation or application of the knowledge. In contrast to the knowledge funnel, the action cycle represents activities needed to apply the knowledge, taking into account the context in which the knowledge is to be used.

Continuing professional development (CPD) is the principal means by which healthcare professionals continue to gain, improve, and broaden the knowledge and the skills they need to provide patient-centered care [23]. In 2008, 93.4% of the 17,758 physicians in the Province of Quebec, Canada, had followed CPD activities or workshops in the previous year [24]. In the United Kingdom, to maintain registered status, nurses are required to undertake a minimum of 35 hours of CPD activities every three years. CPD is therefore likely to be a key intervention for translating SDM into clinical practice [22]. Indeed, results from a recently published Cochrane review of interventions that improve the adoption of SDM by healthcare professionals suggest that both training healthcare professionals and developing patient-mediated interventions, such as patient decision aids, are important for implementing SDM in clinical practice [25]. The review did not inventory CPD programs available for translating SDM into clinical practice.

In summary, CPD can be considered an important KT intervention by virtue of its potential to expand clinicians' adoption of best practices, including the techniques needed for SDM to occur in primary care [22]. However, to remain relevant, CPD must adapt to the ever-changing needs of health professionals, patients, and society [26]. Consequently, our project seeks to increase the current knowledge base of CPD programs and related activities that target translating SDM into primary care clinical practices in diverse healthcare systems. More specifically, this international collaboration will bring together the expertise and the resources needed to develop an interdisciplinary research team dedicated to the study of translating SDM into primary care through effective CPD. Its specific objectives are to develop a collaborative research network; to inventory CPD programs and related activities that seek to translate SDM into clinical practice; to critically appraise the CPD programs identified and review their effects on fostering the practice of SDM; and to identify knowledge gaps in order to generate a research agenda.

## Methods

### Participants

This collaborative project will be developed by an interdisciplinary team composed of researchers from Canada, France, Germany, Switzerland, the United Kingdom, and the United States. Ongoing research activities may cause researchers from countries not yet represented to join the project. Canadian team members will be responsible for coordinating the study, fostering communication among members of the international team, coordinating the environmental scan, inventorying CPD programs and their critical appraisals, and hosting the final workshop. Team members from other countries will provide expertise in implementing SDM through CPD, sharing their experiences and standpoints on the problems and/or challenges involved in this process. They will also help build the inventory of CPD programs for translating SDM in clinical practice and contribute to the research agenda.

### Research activities

#### Environmental scan

An environmental scan is an efficient, organised means for an institution to collect information about its internal and external surroundings [27-29]. Continuing professional educators can also use a scan to identify current and potential learning needs and trends. Conducting a scan thus distinguishes areas in need of improvement, identifies the resources necessary to make those improvements, and ultimately, enhances decision making. In this study, we will perform an environmental scan to identify information about effective CPD programs and related activities for translating SDM into primary care, and to analyse the gaps in the knowledge base.

The literature describes various methodologies and sources for collecting and analysing information for an environmental scan [28]. With help from our research team network and a private firm that specialises in business intelligence and strategic watches, we will begin by identifying professional organisations, academic institutions, and experts in the fields of CPD and SDM. We will contact each one individually and--because we plan to favour sensitivity--inquire about any SDM training programs and/or activities, any published or unpublished evaluations of these programs and/or activities, and any other organisations or experts that may help us to find as many SDM training programs and/or activities as possible.

After having identified SDM training programs and/or activities, we will contact CPD organisations (planners and providers) and invite them to participate in a semi-structured interview. This interview will focus on the organisation of CPD activities geared towards fostering SDM in clinical practice and will be modified in light of interviewees' responses following the first step of the environmental scan.

#### Identification of eligible CPD programs

We understand a CPD activity to be an educational activity that serves to maintain, develop, or increase the knowledge, skills, and professional performance of a licensed healthcare professional who provides services to patients, the public, or the profession (*e.g*., educational meetings and material, audit and feedback, academic detailing) [30]. In this project, a CPD program in SDM is defined of a set of procedures that links clients' needs (healthcare professionals' need to be trained in SDM), activities (a given educational activity), necessary resources (human and material), and immediate and long-term outcomes (licensed healthcare professionals sharing decisions with their patients, and patients' health outcomes). Such a program must comprise at least one CPD activity whose aim is to maintain, develop, or increase the knowledge, skills, and professional performance used by licensed healthcare professionals to share decisions with their patients in a given clinical context.

We expect to identify CDP programs in SDM that meet our inclusion criteria and include at least one CPD activity. It is also possible that we will identify single, isolated SDM CPD activities that are not part of a CPD program in SDM. For each eligible CPD program and/or activity identified, we will ask authors to provide material and a published or unpublished description. All programs and/or activities thus identified will be included in the inventory, independent of the language in which the material was written. A private firm and team members will consider the merits of translating the material into English for our critical appraisal. This work will lay the foundation for the initial inventory, and will ensure that the relevant literature has been appraised and evaluated.

#### Critical appraisal

For each eligible CPD program and/or activity included in the initial inventory, two reviewers (members of the research team) will independently extract characteristics of the SDM CPD program and related activities using a standardised data extraction form. Inspired by the Workgroup for Intervention Development and Evaluation Research (WIDER) reporting guidelines for behavioural interventions, this form will be discussed with all team members and will be adapted to the needs of the study [31]. The two reviewers' extractions will be compared and disagreements resolved through consensus or appeal to the principal investigator. Findings will be entered into a matrix to facilitate comparing the performance of various CPD activities with respect to the characteristics of interest.

Characteristics that will be extracted include the following: identifiers of the training activity (*e.g*., title, authors, year, country, language); types of healthcare professionals targeted (*e.g.*, physicians, nurses, social workers, health psychologists); accreditation and provision of continuing medical education/CPD credits by an official continuing medical education/CPD organisation; objectives of the program; level of the Kirkpatrick model of educational outcomes addressed by the study (*e.g*., reaction, learning, behaviour, results) [32]; essential elements of the integrated model of SDM addressed by the study; mode of delivery (*e.g*., on-line, on site); instructional methods (*e.g*., didactic lectures, workshops, case studies, demonstrations); material available (*e.g*., videos, card games, decision support tools, simulated patients, trainer and/or trainee booklets); duration and frequency of the program; the human and material resources needed to conduct the program; the program's estimated cost; methods and tools to assess how the program impacts participants; empirical data about the efficacy of the program; the transferability of the program to other healthcare professionals and contexts; and updates, modifications, and revisions.

#### Inventory of CPD programs for translating SDM in clinical practice

A summary of each CPD program will be accessible online. Each summary will include the title, the author, the author's website, and other pertinent information.

#### Consensus meeting

Results from the environmental scan and the critical appraisal will be synthesized and the empirical data used to facilitate discussions and identify knowledge gaps during a two-day workshop to be attended by the members of the international collaboration in Quebec City, Canada. This consensus meeting will be critical to distilling the information. When information is presented in a straightforward and precise manner, it becomes possible for experts to assimilate the concepts, discuss the results, and draw logical conclusions. The goal is that research team members will share their unique knowledge and perspectives on translating SDM into primary care through effective CPD and will reach unanimous agreement on the topics discussed. The consensus meeting will be led by a facilitator who is not a team member. This will allow a neutral party to moderate discussions and will ensure that participants respect the time allotted for each topic. Team members will be expected to achieve consensus regarding the gaps in the knowledge and the elements to include in the research agenda. We will produce a brief report summarizing the outcomes of the consensus meeting. The last step of our project goes beyond our research team and involves validating a summary of the study by CPD stakeholders through electronic communication.

#### Ethical considerations

The representatives of CPD organisations whom we will interview will be asked to complete consent forms. Ethical approval for the project was received from the Research Ethics Board Committee of the Centre Hospitalier Universitaire de Québec (CHUQ) on 21 June 2010.

## Discussion

CPD is an important KT intervention that has the potential to promote clinicians' adoption of the most effective practices, including the practices needed for SDM to occur in primary care [22]. An international and interdisciplinary group funded by the Canadian Institutes of Health Research (CIHR) has been created with the purpose of increasing the current knowledge base of CPD programs and activities to translate SDM into primary care in different healthcare systems. Through ongoing exchanges among team members, various perspectives on problems and challenges associated with implementing SDM through CPD in primary care will be made evident. It will then be possible to identify issues related to this important research question. Although some international collaboration has been initiated, there are currently no coordinated efforts to enhance international research in this field.

The environmental scan performed in this study will help determine the existing knowledge base regarding effective CPD for translating SDM into primary care. It will make it possible to identify the individuals or groups initiating CPD activities, the content and quality of CPD training, the strategies and means of conducting CPD training, and the impact of CPD training on fostering SDM. The knowledge acquired from this research will allow us to better understand gaps in the knowledge and will determine which research questions to pursue. Any bias in the interpretation of the environmental data scan [28] is likely to be minimized by the diversity of the perspectives of our multidisciplinary team members.

We acknowledge that a wide range of interventions at various levels in healthcare systems, within organisations, and with patients and healthcare professionals is needed for SDM to be translated into primary care. Given that CPD is such an effective KT intervention, however, we argue that CPD interventions will be essential to causing SDM to enter the action cycle of the knowledge-to-action process. Thus, this project has the potential to produce transformative advances in the implementation of SDM in clinical practice and in the transformation of CPD itself as an effective KT intervention [22].

## Competing interests

The authors declare that they have no competing interests.

## Authors' contributions

All authors collectively drafted the study protocol and approved the final manuscript. FL is its guarantor.
